# Identification and Functional Characterization of a Cold-Related Protein, BcHHP5, in Pak-Choi (*Brassica rapa* ssp. *chinensis*)

**DOI:** 10.3390/ijms20010093

**Published:** 2018-12-26

**Authors:** Jin Wang, Feiyi Huang, Xiong You, Xilin Hou

**Affiliations:** 1 State Key Laboratory of Crop Genetics and Germplasm Enhancement/Key Laboratory of Biology and Germplasm Enhancement of Horticultural Crops in East China, Ministry of Agriculture/Engineering Research Center of Germplasm Enhancement and Utilization of Horticultural Crops, Ministry of Education, Nanjing Agricultural University, Nanjing 210095, China; 2017204023@njau.edu.cn (J.W.); hfy@njau.edu.cn (F.H.); 2 College of Sciences, Nanjing Agricultural University, Nanjing 210095, China

**Keywords:** heptahelical protein 5, cold-related, virus-induced gene silencing, Pak-Choi (*Brassica rapa* ssp. *chinensis*)

## Abstract

In plants, heptahelical proteins (HHPs) have been shown to respond to a variety of abiotic stresses, including cold stress. Up to the present, the regulation mechanism of HHP5 under low temperature stress remains unclear. In this study, *BcHHP5* was isolated from Pak-choi (*Brassica rapa* ssp. *chinensis* cv. *Suzhouqing*). Sequence analysis and phylogenetic analysis indicated that *BcHHP5* in Pak-choi is similar to *AtHHP5* in *Arabidopsis thaliana*. Structure analysis showed that the structure of the BcHHP5 protein is relatively stable and highly conservative. Subcellular localization indicated that *BcHHP5* was localized on the cell membrane and nuclear membrane. Furthermore, real-time quantitative polymerase chain reaction (RT-qPCR) analysis showed that *BcHHP5* was induced to express by cold and other abiotic stresses. In Pak-choi, *BcHHP5*-silenced assay, inhibiting the action of endogenous *BcHHP5*, indicated that *BcHHP5*-silenced might have a negative effect on cold tolerance, which was further confirmed. All of these results indicate that *BcHHP5* might play a role in abiotic response. This work can serve as a reference for the functional analysis of other cold-related proteins from Pak-choi in the future.

## 1. Introduction

At present, adverse environmental conditions seriously affect the growth and development of plants. In particular, abiotic stresses, such as cold, drought, and heat have significant adverse effects on agricultural production [1,2]. Tolerance to abiotic stresses is critical to plant growth and development, as plants cannot move to avoid adverse environments. Chilling (low temperatures below 0 °C) and freezing (low temperatures below 0 °C) can greatly reduce crop yields [3]. Plants use complex interconnected signaling networks to address low temperature non-frozen abiotic stresses [4]. Temperature is one of the main environmental factors that limit plant growth and development. In particular, cold stress is a serious threat to the sustainability of crop yields. Plants face a complex environment and the mechanism of regulation of plant responses to abiotic stresses remains to be discovered. Heptahelical protein 1 (HHP1) might act as a negative regulator of abscisic acid (ABA) stress [5]. Since heptahelical protein 5 (HHP5) and heptahelical protein 1 (HHP1) all belong to the *HHP* gene family, we wonder if they share a similar function.

Pak-choi (genus *Brassica*) and *Arabidopsis* (a model species) belong to the Brassicaceae family and they have a close relationship [6]. The expression behaviors of some cold stress-related genes in *Arabidopsis*, such as *CBFs* (c-repeat-binding factor), *CORs* (cold-regulated), and *HHPs*, have been extensively investigated [1,7,8,9]. These genes have been shown to play a major role in the response to environmental stresses [10]. In *Arabidopsis*, HHP1 acts as a bridge for the ABA regulation of cold signaling components [11,12]. Although functional studies of *Arabidopsis* HHP have been reported, few papers on HHP in Pak-choi have ever been seen in literature.

Pak-choi (*Brassica rapa* ssp. *chinensis*) has a long history of cultivation in wide areas of Asia, particularly in the middle and lower reaches of the Yangtze River in China. The research on the response mechanism of Pak-choi to abiotic stresses is very important [13]. In this study, the *HHP5* gene was cloned from Pak-choi (*Brassica rapa* ssp. *chinensis* cv. *Suzhouqing*) and named as *BcHHP5* (CabbageG_a_f_g000167). We analyzed the sequence structure and studied the evolutionary position through phylogenetic trees, the SOMPA online tool, and the SWISS-MODEL online tool. Furthermore, we found that the homologous relationship of *BcHHP5* and *AtHHP5* is close, conservatively. We analyzed the phylogenetic patterns and conservative motifs of *BcHHP5* to explore whether *BcHHP5* and *AtHHP5* have similar structures and functions. Subcellular localization assay indicated that *BcHHP5* localizes on the cell membrane and nuclear membrane. In addition, we used the real-time quantitative PCR (RT-qPCR) to analyze the expression patterns of *BcHHP5* under various abiotic stresses. We tested viral-induced *BcHHP5*-silencing, which reduced the response to cold tolerance. In conclusion, the present study suggests that *BcHHP5* might play a role in cold stress response, and this might also help to further reveal the cold tolerance mechanism in Pak-choi.

## 2. Results

### 2.1. Isolation and Multiple Sequence Alignment of BcHHP5

**Figure 1:**
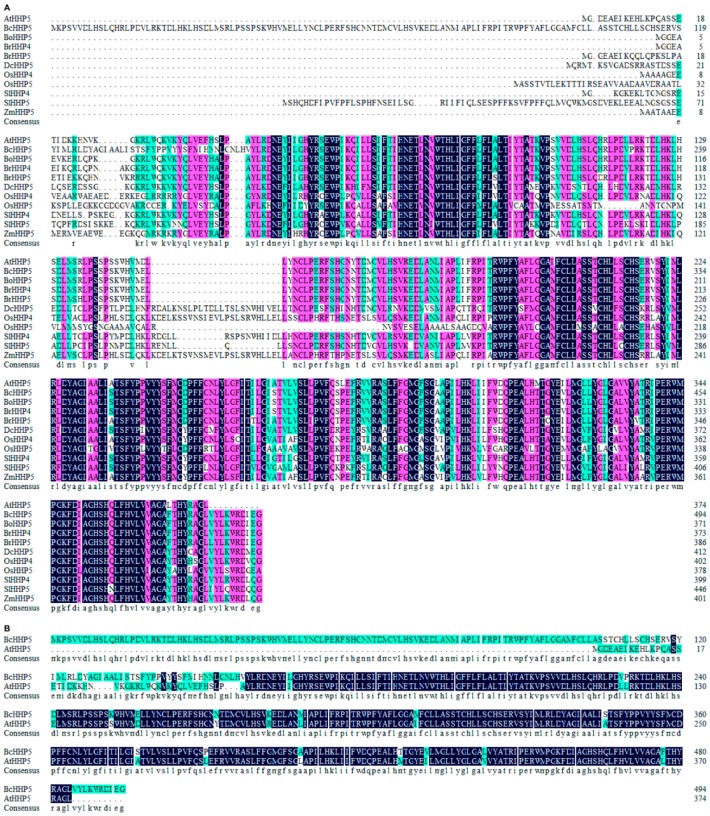
Multiple sequence alignments corresponding to the encoded amino acid sequences on the 11 heptahelical protein (*HHP*) genes. (**A**) In this study, HHPs in Pak-choi (BcHHPs) were isolated and other crop proteins were also selected from GenBank; (**B**) BcHHP5 and *Arabidopsis thaliana* heptahelical proteins (AtHHP5) were isolated in the study and selected from GenBank. Less conservative, highly conservative, and perfectly matched residues are represented by green boxes, pink boxes, and black boxes, respectively.

In the study, we isolated the *HHP5* gene in Pak-choi and named it as *BcHHP5*. The conserved domains of 11 *HHP* genes were visualized through the NCBI online platform (https://www.ncbi.nlm.nih.gov/). By the multiple sequences alignment, it was revealed that those sequences of *HHPs* had some similarity (Figure 1A). Further, we found that the sequences of *BcHHP5* and *AtHHP5* had 63.23% similarity and similar homology (Figure 1B). The similarities of the sequences were further analyzed by multiple sequence alignment. These results showed that the two sequences had some identities in their regulatory regions. It was determined whether the expression pattern of *BcHHP5* was a response to cold stress in Pak-choi. The gene accession numbers are as follows: *AtHHP5* (At4g38320), *BcHHP5* (CabbageG_a_f_g000167), *BoHHP5* (Bol028895), *BrHHP4* (Brara.A00124.1), *BrHHP5* (Brara.K01231.1), *DcHHP5* (DCAR_021463), *OsHHP4* (LOC_Os03g13040.1), *OsHHP5* (LOC_Os03g10300.1), *SlHHP4* (Solyc02g092230.2.1), *SlHHP5* (Solyc03g043930.2.1), and *ZmHHP5* (GRMZM2G161780_T01).

### 2.2. Phylogenetic Analysis and Motif Analysis

**Figure 2:**
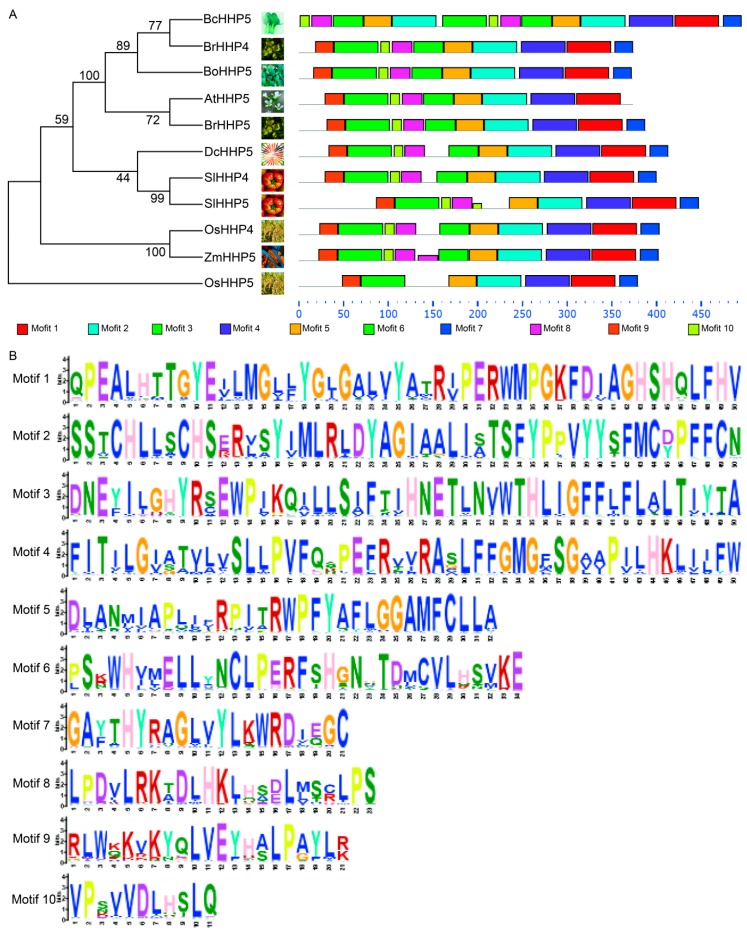
Conserved motifs in *HHP*. (**A**) Phylogenetic tree, conserved motifs, and their distribution in each *HHP* gene and the corresponding combined P-values; (**B**) the amino acid sequence of each motif. The font size means the frequency of the corresponding amino acid.

MEGA 6.0 software [14] was used to construct a phylogenetic tree by the Neighbor-Joining (NJ) method and the fully predicted HHP amino acid sequence of the following: *At*, *Arabidopsis thaliana*; *Bo*, *Brassica oleracea*; *Bc*, *Brassica campestris*; *Br*, *Brassica rapa*; *Dc*, *Daucus carota*; *Os*, *Oryza sativa*; *Sl*, *Solanum lycopersicum*; and *Zm*, *Zea mays* (Figure 2A). Phylogenetic analysis of BcHHP5 and *Arabidopsis* HHP5 were constructed. We found that BcHHP5 showed greater similarity to AtHHP5. To search for potentially conserved sequences in the complete amino acid sequence of the HHP protein, we used Multiple EM for Motif Elicitation (MEME) (http://meme-suite.org/) [15] with default parameters, except that the number of motifs was set to 10. Conserved motifs for all HHP proteins are shown in Figure 2B by using the MEME online tool (http://meme-suite.org/tools/meme). At the same time, we determined 10 default motifs by MEME analysis. There were eight HHP proteins that contained 10 motifs, one HHP protein (SlHHP5) that contained nine motifs, one HHP protein (OsHHP5) that only contained seven motifs, and the BcHHP5 protein encompassed 10 motifs, which might be due to certain loss events during the evolution of the species. In addition, members of the same phylogenetic tree clades had similar motifs organization in terms of the gene length or the number of motifs [16].

### 2.3. Structure Analysis of BcHHP5 Protein

**Figure 3:**
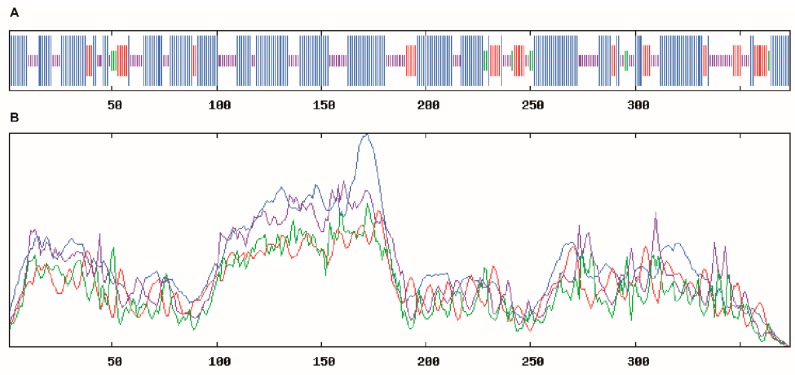
Secondary structural analysis of the BcHHP5 Protein. (**A**) Comparison of the secondary structures of BcHHP5 protein; (**B**) helix, turn, strand, and coil were represented with blue, red, green, and purple lines, respectively.

**Figure 4:**
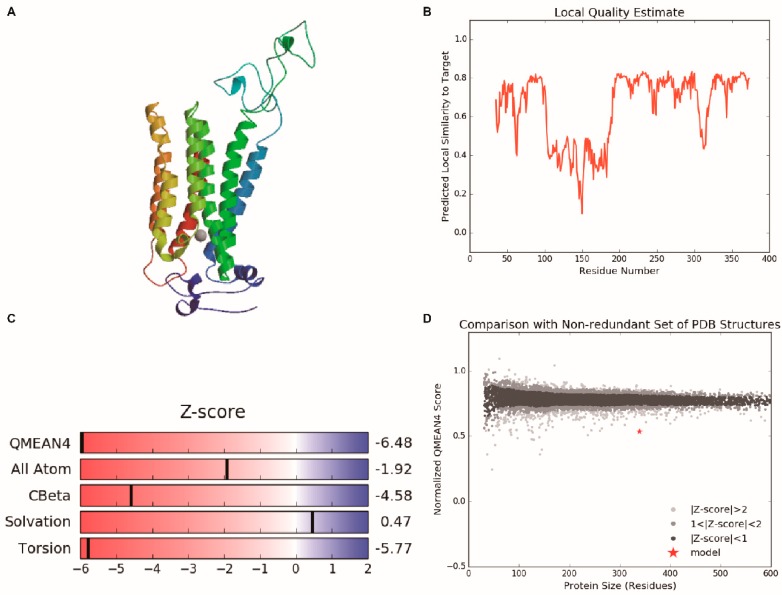
3D structural analysis of BcHHP5 Protein. (**A**) The predicted 3D structure of BcHHP5 protein; (**B**) local quality estimate of BcHHP5 protein; (**C**) global quality estimate of BcHHP5 protein; (**D**) a comparison of non-redundant sets with the Protein Data Bank (PDB) structural model.

Through the SOPMA online tool, the secondary structure of the BcHHP5 protein in Pak-choi was analyzed and predicted. The secondary structure analysis revealed that the BcHHP5 protein was mainly composed of free-curl, α-helix, and β-sheet, which is consistent with previous studies on BcHHP5 protein structure. The results of the SOPMA provided the following information: with the alpha helix (Hh): 261, 52.73%; 3_10_ helix (Gg): 0, 0.00%; pi helix (Ii): 0, 0.00%; beta bridge (Bb): 0, 0.00%; extended strand (Ee): 68, 13.74%; beta turn (Tt): 25, 5.05%; bend region (Ss): 0, 0.00%; random coil (Cc): 141, 28.48%; ambiguous states (?): 0, 0.00%; other states: 0, 0.00%; parameters, window width: 17; similarity threshold: 8; number of states: 4 (Figure 3). The secondary structure might serve to protect the BcHHP5 protein from certain endonuclease damage [17]. In addition, using the SWISS-MODEL online tool, the three-dimensional structural model of the BcHHP5 protein was predicted [18]. The sequence identity was as follows: 42.96%; oligo-state: hetero-trimer; method: X-ray, 2.40 Å; sequence similarity: 0.39; range: 151–493; coverage: 0.55; description: human adiponectin receptor 5 (Figure 4). It was noteworthy that α-helix occurred predominantly in the structure of the BcHHP5 protein and there were amino acid residues that were predicted to be exposed, which revealed that BcHHP5 might not be globular. The results showed that the structure of the BcHHP5 protein is relatively stable, which demonstrates high conservation.

### 2.4. Transmembrane Domain, Signal Peptide, Hydrophilicity, and Subcellular Localization Prediction Analysis

**Figure 5:**
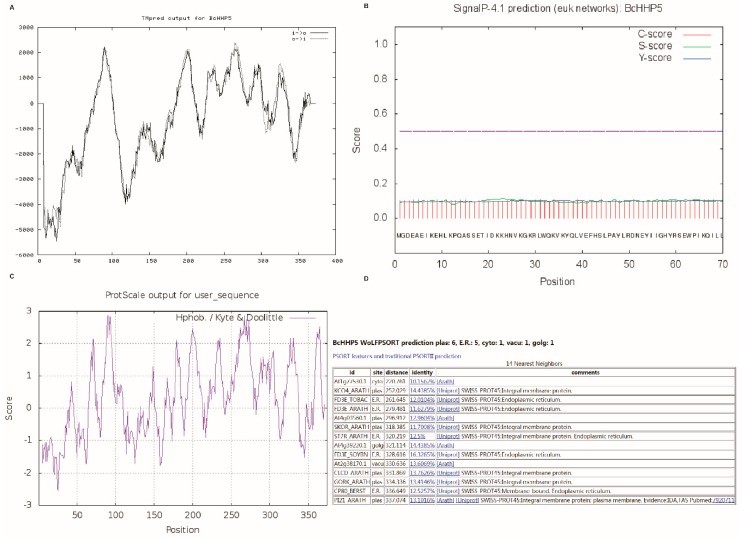
Related information of the BcHHP5 protein. (**A**) Transmembrane domain of the BcHHP5 protein; (**B**) signal peptide of BcHHP5 protein; (**C**) hydrophilicity of the BcHHP5 protein; and (**D**) subcellular localization prediction analysis of the BcHHP5 protein.

As shown in Figure 5A, by using important TM-segments, we obtained a strongly preferred model of the inner N-terminus with six powerful transmembrane helices with a total score of: 11291 from length score orientation, (1) 82-101 (20) 2220 i-o; (2) 190-212 (23) 2131 o-i; (3) 228-244 (17) 1376 i-o; (4) 256-274 (19) 2407 o-i; (5) 287-306 (20) 1568 i-o; and (6) 317-337 (21) 1589 o-i. We found that the BcHHP5 protein is a hydrophilic protein (Figure 5B). We concluded by reporting the maximum of the three scores. The following two scores are displayed: the average S-score of possible signal peptides (from position one to the position immediately before the maximum Y-score), and the D score (discrimination score), which is the weighted average of the average S and the maximum value. The Y score was used to distinguish the scores of signal peptides from non-signal peptides (Figure 5C). For non-secreted proteins, all fractional values represented in the SignalP output should be very low (possibly close to a negative target of 0.1). According to WOLF PSORT (https://wolfpsort.hgc.jp/), the final localization score of BcHHP5 was 9.0 (KNN = 14), and subcellular location predicted by Nuc-PLoc might be the plasma membrane and the endoplasmic reticulum (Figure 5D). These results indicate that BcHHP5 protein is a hydrophobic protein with no signal peptide and no transmembrane region.

### 2.5. Localization Analysis of BcHHP5 Protein

**Figure 6:**
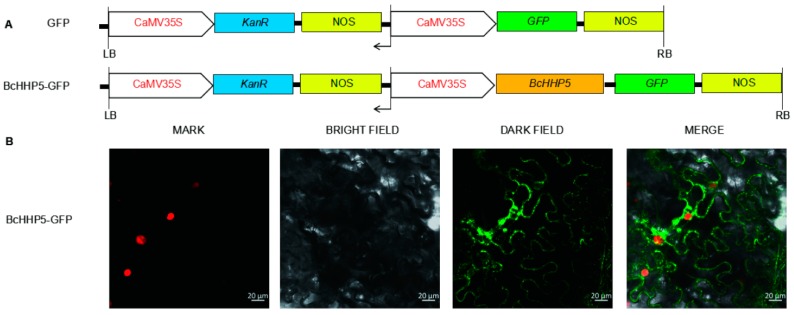
Localization analysis of the BcHHP5 protein. (**A**) Schematic representation of the construction of *35S: GFP* and *35S: BcHHP5-GFP*; (**B**) schematic representation of the transient expression of *35S: GFP* and *35S: BcHHP5-GFP* fusion proteins in tobacco leaves (20 μm scale).

In order to obtain transient over-expression in tobacco leaves, the subcellular localization of *35S: BcHHP5-GFP* fusion was detected by using the *Agrobacterium* infiltration method (Figure 6A). On the cell membrane and the nuclear membrane, we were able to observe the GFP fluorescence of the *35S: BcHHP5-GFP* fusion protein. At the same time, the fluorescence of *35S: GFP* was observed in both the nucleus and the cytoplasm (Figure 6B). These results suggest that *BcHHP5* might target the cell membrane and the nuclear membrane. Because transcription factors are often localized on the nucleus or the nuclear membrane, we suspect that *BcHHP5* might be a transcription factor [19].

### 2.6. Expression Levels of the BcHHP5 Gene in Pak-Choi

**Figure 7:**
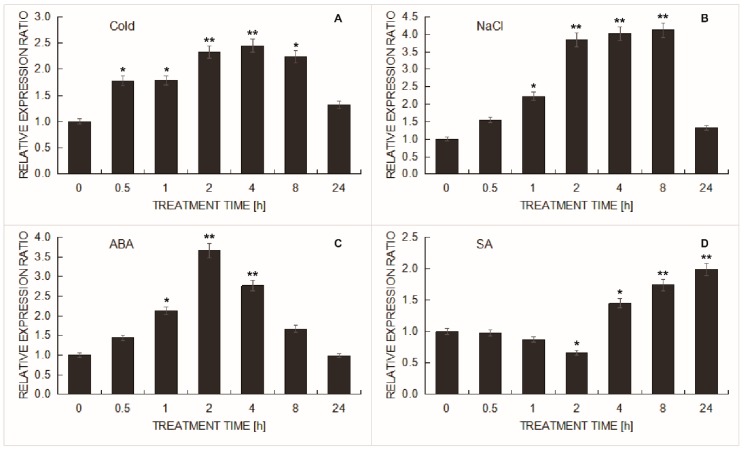
The expression patterns of *BcHHP5* in Pak-choi under abiotic stress were analyzed by RT-qPCR. (**A**) Cold treatment, *BcHHP5* expression; (**B**) salt treatment, *BcHHP5* expression; (**C**) ABA treatment, *BcHHP5* expression; (**D**) salicylic acid (SA) treatment, *BcHHP5* expression. *: 0.01 < *P* < 0.05, **: *P* < 0.01.

To investigate the expression levels of the *BcHHP5* gene for different abiotic stresses, we performed RT-qPCR assay on the *BcHHP5* gene for analysis. As shown in Figure 7, under the abiotic stress, the *BcHHP5* gene showed various expression patterns. With the cold stress treatment, the relative transcription level of *BcHHP5*, compared with the control, increased and reached its maximum value at 4 h and then decreased rapidly (Figure 7A). With NaCl stress treatment, the *BcHHP5* relative expression level increased slowly and reached its maximum value after 8 h, after which it rapidly decreased (Figure 7B). With ABA stress treatment, the relative expression level of *BcHHP5* significantly increased and reached its maximum value after 2 h (Figure 7C). However, with salicylic acid (SA) stress treatment, the relative transcription level of *BcHHP5* slightly decreased and then increased (Figure 7D). The above data indicates that expression of *BcHHP5* was affected by these stress treatments. Moreover, these data also imply that the induction kinetics of *BcHHP5* in cold stress is similar to that in ABA stress. With the cold stress treatment or ABA stress treatment, the expression of *BcHHP5* messenger ribonucleic acid (mRNA) in leaves showed a significant increase in 4 h, indicating that *BcHHP5* might be cold-induced and involved in cold treatment and ABA treatment co-reactions. Furthermore, since the expression of *BcHHP5* did not increase rapidly after temperature drops but was induced 2 h after cold stress treatment, we speculate that *BcHHP5* is associated with translational arrest in cold stress. The expression levels of *BcHHP5* relative to *BcACTIN* were digitized using 2**^−^**^ΔΔ^*^C^*^t^: ΔΔ*C*T = Δ*C*T_stress sample_ − Δ*C*T_un__-stressed sample_, Δ*C*T = CT_target_ − CT_BcACTIN_. Each column represents the mean and the standard deviation of the values represented by three repetitions. Significant differences between treatments were as follows, respectively (ANOVA calculated using SPSS) *: 0.01 < *P* < 0.05, **: *P* < 0.01. Each set of data consisted of the mean ± SEM of three replicates and independent experiments.

### 2.7. Virus-Induced BcHHP5 Silencing Reduced Response to Cold Tolerance

**Figure 8:**
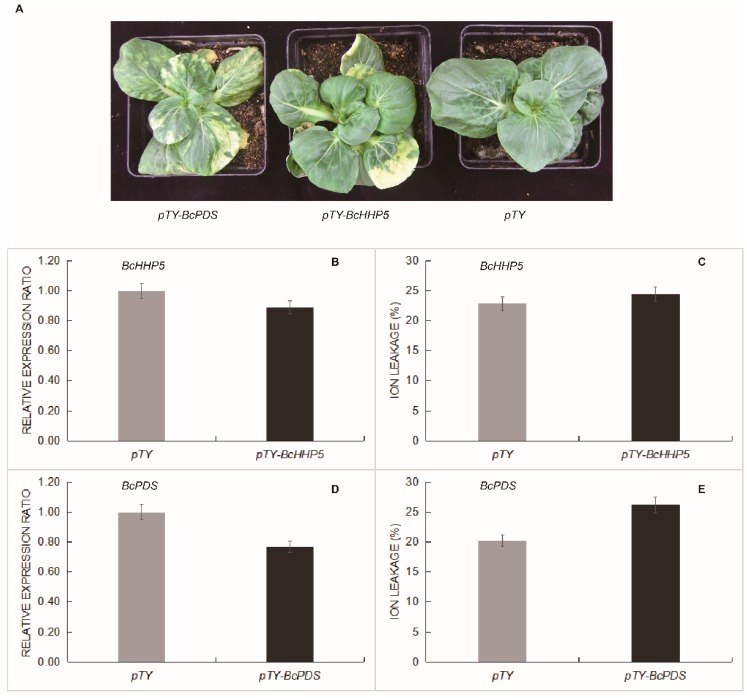
BcHHP5 silencing in Pak-choi. (**A**) The Pak-choi leaf phenotype (2.5 cm scale) after *BcHHP5*-silencing test; (**B**) relative expression of *BcHHP5*; (**C**) ion leakage of Pak-choi leaves; (**D**) relative expression of *BcPDS*; (**E**) ion leakage of Pak-choi leaves.

In Pak-choi, we used the turnip yellow mosaic virus-induced gene silencing (TYMV-VIGS) to get *BcHHP5-*silence. In order to further functionally study the role of *BcHHP5* cold tolerance, we constructed corresponding vectors and bombarded them with a particle gun, then bombarded these vectors into the Pak-choi leaves and subsequently cultivated them. Three weeks later, we observed the phenotype of Pak-choi after the bombardment of the three vectors (*pTY*, *pTY-BcHHP5*, and *pTY-BcPDS*), respectively, and further extracted the total RNA from the positive plant leaves. As shown in Figure 8A, after *BcHHP5*-silenced, we observed the phenotype of Pak-choi leaves, which showed green loss. To demonstrate whether the silencing effect reached the expected level, we used RT-qPCR assay to detect the relative expression levels of *BcHHP5* and *BcPDS* in positive plants. We found that *BcHHP5* expression was significantly decreased in *BcHHP5*-silenced plants, and its numerical decline rate was almost 10%. At the same time, in *BcPDS*-silenced plants, *BcPDS* expression was significantly decreased, and the numerical decline rate was almost 12% (Figure 8B,D). Consistently, ion leakage of *pTY-BcHHP5* plants was increased compared with *pTY* plants (Figure 8C), and these *pTY-BcPDS* plants increased more ion leakage compared with the *pTY* plants (Figure 8E). Taken together, these results suggest that virus-induced *BcHHP5* silencing attenuates the response function in cold signaling.

## 3. Discussion

Low temperature is the main environmental pressure that affects plant survival. In cold conditions, it can enhance the cold tolerance of certain plants, and this process is called cold acclimation. Extensive biochemical reactions occur in plants, enabling them to grow under certain conditions of low temperatures [20]. These biochemical reactions are regulated by different expression levels of many cold-related genes [1]. In *Arabidopsis*, the HHP1 protein was identified and was shown to be homologous to the other four HHP proteins (HHP2, HHP3, HHP4, and HHP5) [9]. In this study, we successfully isolated and functionally identified the HHP5 protein in the Pak-choi cultivar *Suzhouqing*, and we named it BcHHP5. We identified that *BcHHP5* might respond to cold stress. By analyzing the sequence structure and evolutionary position through phylogenetic trees, we found that the homologous relationship of *BcHHP5* and *AtHHP5* is close, and the *BcHHP5* sequence is highly conservative. This suggests that these genes might have some similar functions in some aspects. Studies of chill shock (4–10  °C) and freeze shock (<0  °C) stresses in a number of different crops have resulted in a similar cold response regulation and signaling cascade [21]. The *BcHHP5* gene was selected and induced by the above four abiotic stresses, respectively. This study also demonstrated that BcHHP5 localizes on the cell membrane and nuclear membrane [22]. We further investigated *BcHHP5* functions by silencing it in Pak-choi.

Many plants—not model crops—can grow in adverse environments because their unique genetic resources allow them to adapt to natural disadvantages [23]. As one of the most popular fresh vegetables, Pak-choi often undergoes a variety of adverse stresses in its life span [24]. When HHP1 is exposed to various stresses (cold, ABA, and salt stress), its relative expression levels increase [11]. Through experimental studies, we found that *BcHHP5* expression showed a similar relative expression pattern and induction kinetics under prolonged hypothermia or ABA treatment (Figure 7). In addition, cold (salt) stress also affected the relative expression pattern of *HHP* genes to varying degrees [9]. The reduced response to cold tolerance in *BcHHP5*-silenced Pak-choi plants (Figure 7) suggests that *BcHHP5* might be a low temperature regulator.

In summary, *BcHHP5* might be a low temperature regulator in Pak-choi and might respond to low temperature stress. *BcHHP5* is a protein that is localized on the cell membrane and the nuclear membrane. This is the first study on the *BcHHP5* in the regulation of cold response. The study might help to further elucidate the regulatory mechanisms of plants in low temperature stress, not only in Pak-choi, but also in other related species. In addition, other cold-related genes also exist in Pak-choi, and their functions need to be further explored.

## 4. Material and Methods

### 4.1. Cloning, Multiple Sequence Alignment, and Phylogenetic Analysis

The leaves of Pak-choi were collected at their five-leaf stage, and then the reverse transcription and synthesis of the first strand complementary DNA (cDNA) was carried out. cDNA was used as a template, and primers of BcHHP5 (Table 1) were used as primers to perform PCR amplification. The amplification procedure was as follows: 98 °C, 2 min; 35 cycles (98 °C, 30 s; 57 °C, 30 s; and 72 °C, 1 min) and was extended at 72 °C for 10 min. Subsequently, we performed total RNA extraction, cDNA synthesis, and PCR amplification, respectively [25]. Amplification primers were used to amplify the open reading frames (ORFs) of tested genes, and then the target fragment was constructed onto the pMD19-T vector (Takara, Beijing, China) and finally sequenced separately. Based on the outlined procedure, multiple sequence alignments were performed by using DNAMAN software. A phylogenetic tree was generated by using the full-length protein sequences of the listed species (Table 2), and the bootstrap values were evaluated with 1000 replicates by the adjacent ligation method using MEGA 6.0 software.

### 4.2. Plant Materials, Growing Environment, and Adversity

Pak-choi was grown in a greenhouse environment (light: 22 °C for 16 h; dark: 18 °C for 8 h). Seedlings were used during the five-leaf period, and all treatments (cold, salt, ABA, and SA) were carried out under hydroponic conditions. The Pak-choi cultivar *Suzhouqing*’*s* cold tolerance is better than other varieties. Therefore, it is more suitable for VIGS tests. We harvested the Pak-choi leaves, froze them in liquid nitrogen, and stored them at -80 °C with three biological replicates in order to analyze the relative expression of *BcHHP5* under different stress treatments. The sequences of VIGS are listed in Table 3. These plants, which were bombarded with the *pTY*, *pTY-BcHHP5*, and *pTY-BcPDS* plasmids, respectively, were grown in the same environment (light: 22 °C for 16 h; dark: 18 °C for 8 h). After 15 days, we began to observe the phenotype of the leaves while collecting the leaves for RNA extraction. In addition, we collected Pak-choi samples in cold stress for RT-qPCR analysis.

### 4.3. BcHHP5 Protein Structural Analysis in Pak-Choi

The structure of the BcHHP5-encoded protein in Pak-choi was predicted through the SOMPA (http://npsa-pbil.ibcp.fr/cgi-bin/npsa_automat.pl?page=npsa_sopma.html) and the SWISS-MODEL (http://sissmodel.expasy.org/) online tools, respectively.

### 4.4. Transmembrane Domain, Signal Peptide, Hydrophilicity, and Subcellular Localization Prediction Analysis

The transmembrane domain, signal peptide, hydrophilicity, and subcellular localization prediction analysis of the BcHHP5-encoded protein in Pak-choi were predicted separately through the online tools at (http://www.ch.embnet.org/software/TMPRED_form.html), (http://www.cbs.dtu.dk/services/SignalP), (http://web.expasy.org/protscale/), and (https://npsa-prabi.ibcp.fr/cgi-bin/npsa_automat.pl?page=/NPSA/npsa_sopma.html), respectively.

### 4.5. Localization Method of 35S: BcHHP5-GFP

We amplified the protein coding region of *BcHHP5* without a stop codon and then fused it with GFP under the CaMV35S promoter. It was then further constructed into the pCambia1302 vector to generate a new fusion vector, the *35S: BcHHP5-GFP*. The *35S: BcHHP5-GFP* plasmid and the *35S: GFP* plasmid were conducted into *Agrobacterium* tumefaciens (strain GV3101) by the freeze-thaw method and were injected into tobacco leaves [26]. Then, the tobacco leaves were stained with marker, a red nuclear dye, to confirm nuclear localization. After 48 h of incubation at 25 °C, we observed whether there was GFP in tobacco leaves with the confocal microscope instrument (Leica, TCS SP2, Wetzlar, Germany).

### 4.6. Cold, NaCl, ABA, and SA Treatments in Pak-Choi

We investigated the expression level of *BcHHP5* with stress treatments by real-time quantitative PCR. The corresponding primers for RT-qPCR, listed in Table 1, were designed by the GenScript Biotech Corp. (Hong Kong, China) (https://www.genscript.com/ssl-bin/app/primer) online tool. At the five-leaf stage, Pak-choi plants were used for RT-qPCR analysis. We obtained plant leaves under different treatment conditions [27]. For low temperature treatment, we placed these plants in a 4 °C constant temperature incubator and controlled it in order to sample at different time periods (0, 0.5, 1, 2, 4, 8, and 24 h). For salt or hormone treatments, plants were housed by hydroponics in a constant temperature incubator at 22 °C. For the salt treatment, 100 mM NaCl was added to the water culture nutrient solution for 0, 0.5, 1, 2, 4, 8, and 24 h, respectively, while for the hormone treatment, 100 μM ABA or 0.1 mM SA was added for 0, 0.5, 1, 2, 4, 8, and 24 h, respectively. In plant hormones, SA (ABA) is negatively (positively) correlated with cold response. Experiments have shown the effect of these plant hormones on cold response [28]. We extracted the total RNA by using an RNA extraction kit (Tiangen, Beijing, China) and using the previously preserved plant tissues. To remove genomic DNA contamination, we used DNase I (Takara, Dalian, China). The first strand of cDNA was synthesized from DNase I-treated (1 μg) total RNA for RT-qPCR by using PrimeScript^TM^ RT kit (Takara, Dalian, China). The cDNA reaction mixture was diluted at a ratio of 1:10 by using EASY Dilution for RT-qPCR solution (Takara, Dalian, China). RT-qPCR assay was performed using a 7500 Fast RT-qPCR system (Applied Biosystems, Foster City, CA, USA) with 2 μL cDNA and 10 μL premix (Tara, Dalian, China). The RT-qPCR assay procedure was as follows: 95 °C for 4 min, then 40 cycles (95 °C 30 s, 58 °C 30 s), and 72 °C for 30 s. We generated a melting curve after each RT-qPCR to determine whether the amplification product was specific. The expression level of *BcHHP5* was calculated by the 2^−ΔΔ*C*T^ method. The Pak-choi *BcACTIN* gene was used as an internal reference [29]. In order to reduce the standard error, the test was repeated three times and the obtained data were averaged.

### 4.7. Physiological Analyses and Data Analysis

In order to test the freezing tolerance of the tested plants, we conducted an ion leakage rate assay [30]. Plant tolerance to various abiotic stresses (including cold) has been estimated based on measurements of stress-induced ion leakage from plants. It has become universally accepted that the more plants are stressed, the more ion leakage they will have [31,32,33]. The seedlings were collected and placed in 15 mL tubes with 5 mL deionized water at 22 °C. The initial ion leakage was measured as S0. Then, the seedlings were shaken (60 rpm) at 22 °C for 15 min and the ion leakage was detected as S1. After that, the seedlings were boiled in a 100 °C water bath for 15 min, shaken (60 rpm) at 22 °C for 1 h, and the ion leakage was detected as S2 [34]. These values (S0, S1, and S2) were measured by the DDS-307A instrument (Shanghai Precision Instrument Co.,Ltd., Shanghai, China). The equation (S1-S0)/(S2-S0) was applied to calculate the ion leakage rate of the collected seedlings.

The data from RT-qPCR experiments were tested by using one-way analysis of variance (ANOVA). Statistical data analysis was performed using SPSS v.19.0 software differences between the control, while the treated plants were analyzed by two-way ANOVA according to Duncan’s test. A single asterisk indicates significant differences (0.01 < *P* value < 0.05) and a double asterisk indicates extremely significant differences (*P* value < 0.01). The ANOVA analysis was conducted by SPSS v.19.0 software. Three biological replicates and three technical replicates were used [35].

### 4.8. Silencing Expression of BcHHP5 Gene in Pak-Choi

We designed a 40 bp specific fragment sequence (an antisense form to form a self-hybrid palindromic oligonucleotide). This sequence was synthesized by the GenScript Biotech Corp. (Hong Kong, China) for the VIGS test. Then, self-hybrid palindromic oligonucleotides were inserted into the *pTY-S* (*pTY*) vector of the turnip yellow mosaic virus-induced gene silencing system (TYMV-VIGS) to form a BcHHP5-silenced construct [36]. The *pTY* empty vector and the *pTY-BcPDS* (Phytoene desaturase) vector were used as negative and positive controls, respectively. Pak-choi cultivars, *Suzhouqing*, were used for VIGS after they were grown for about 4 weeks. The *pTY*, *pTY-BcHHP5*, and *pTY-BcPDS* plasmids (5 μg) were coated on the gold particles and bombarded into the leaves of the plants by a gene bombardment gun (Bio-Rad, PDS1000 / He) [37]. Each time, a gene gun bombarded four plants, and for each test, three replicates of the experiment were performed. In the gene silencing experiment, we first observed the phenotype of the plants three weeks later. When the phenotype appeared, all of these materials (the *pTY*, *pTY-BcHHP5*, and *pTY-BcPDS* plants) were exposed to a low temperature (4 °C) environment for 4 h. Then, we performed the RT-qPCR and the ion leakage tests to detect the gene expression level and ion leakage change of gene-silenced plants and the control in low temperature. We wanted to demonstrate whether low temperature treatment would cause changes in gene expression levels and ion leakage in these gene-silenced plants and the control.

## 5. Conclusions

In this study, the *HHP5* gene was cloned from Pak-choi (*Brassica rapa* ssp. *chinensis* cv. *Suzhouqing*) and was named as *BcHHP5*. We analyzed the sequence structure and studied the evolutionary position through sequence alignment and phylogenetic tree. Sequence alignment analysis showed that *BcHHP5* and *AtHHP5* sequences have a high similarity, while phylogenetic tree analysis showed that the evolutionary relationship between *BcHHP5* and *BrHHP4* is relatively close, which revealed homologous relationship of *BcHHP5* and *AtHHP5* or *BrHHP4* is very close and highly conservative. It concludes that these genes might have some similar functions in some aspects. The expression levels of *BcHHP5* gene were tested under four different treatment conditions (cold, salt, ABA, and SA). Besides, this study also demonstrated that *BcHHP5* was localized on the cell membrane and nuclear membrane, which indicated *BcHHP5* might be a transcription factor. In addition, the BcHHP5-silenced assay indicated that BcHHP5-silenced might have a negative effect on cold tolerance, which was further confirmed. All of these results demonstrate that *BcHHP5* might play a role in abiotic response. This work might serve as a reference for the functional analysis of other cold-related proteins from Pak-choi or other species in the future.

## Figures and Tables

**Table 1:** Primer sequences used in this study.

Primer Name	Primer Sequences (5′-3′)
Cloning	
BcHHP5-F	ATGAAACCCAGCGTTGTTGATC
BcHHP5-R	TCAACATCCTTCAATGTCTCTCCAC
Gateway Cloning	
gBcHHP5-F	GGGGACAAGTTTGTACAAAAAAGCAGG
	CTTCATGAAACCCAGCGTTGTTGATC
gBcHHP5-R	GGGGACCACTTTGTACAAGAAAGCTGGG
	TGTCAACATCCTTCAATGTCTCTCCAC
RT-qPCR	
qBcHHP5-F	CATAATGCTAAGGCTTGACT
qBcHHP5-R	GAAGATGCTGAGGAGGAT
qBcPDS-F	CAAAATGGGGTTTTGGAGGC
qBcPDS-R	TGCAGGACGAGGAGCACTACG
qBcACTIN-F	GTTGCTATCCAGGCTGTTCT
qBcACTIN-R	AGCGTGAGGAAGAGCATAAC

Note: Under line sequences represent gateway adapter sequences.

**Table 2:** HHP protein sequences from some different crops.

Name	GenBank Accession	Protein Sequence
AtHHP5	AT4G38320	MGDEAEIKEHLKPQASSETIDKKHNVKGKRLWQKVKYQLVEFHSLPAYLRDNEYIIGHYRSEWPIKQILLSIFTIHNETLNVWTHLIGFFLFLALTIYTATKVPSVVDLHSLQHRLPDLLRKTDLHKLHSELMSRLPSSPSSWHVMDLLYNCLPERFSHGNYTDMCVLHSVREDLANLIAPLIFRPITRWPFYAFLGGAIFCLLASSTCHLLSCHSERVSYIMLRLDYAGIAALIATSFYPPVYYSFMCDPFFCNLYLGFITILGIATVLVSLLPVFQSLEFRVVRASLFFGMGFSGLAPILHKLIIFWDQPEALHMTGYEILMGLLYGLGAVVYATRIPERWMPGKFDIAGHSHQLFHVLVVAGALTHYRAGL
BcHHP5	CabbageG_a_f_g000167	MKPSVVDLHSLQHRLPDVLRKTDLHKLHSDLMSRLPSSPSKWHVMELLYNCLPERFSHGNNTDMCVLHSVKEDLANMIAPLIFRPITRWPFYAFLGGAMFCLLASSTCHLLSCHSERVSYIMLRLDYAGIAALISTSFYPPVYYSFMIHNNLGNLHVYLRDNEYILGHYRSEWPIKQILLSIFTIHNETLNVWTHLIGFFLFLALTIYTATKVPSVVDLHSLQHRLPDVPRKTDLHKLHSDLMSRLPSSPSKWHVMELLYNCLPERFSHGNNTDMCVLHSVKEDLANMIAPLIFRPITRWPFYAFLGGAMFCLLASSTCHLLSCHSERVSYIMLRLDYAGIAALISTSFYPPVYYSFMCDPFFCNLYLGFITILGISTVLVSLLPVFQSPEFRVVRASLFFGMGFSGAAPILHKLIIFWDQPEALHTTGYEVLMGLLYGLGALVYATRIPERWMPGKFDIAGHSHQLFHVLVVAGAFTHYRAGLVYLKWRDIEGC
BoHHP5	Bol028895	MGGEAEVKERLQPKGKRLWQKVKYQLVEYHALPAYLRDNEYILGHYRSEWPIKQILLSIFTIHNETLNVWTHLIGFFLFLALTIYTATKVPSVVDLHSLQHRLPDLLRKTDLHKLHSDLMSRLPSSPSKWHVMELLYNCLPERFSHGNSTDMCVLHSVKEDLANMIAPLIFRPITRWPFYAFLGGAMFCLLASSTCHLLSCHSERVSYIMLRLDYAGIAALISTSFYPPVYYSFMCDPFFCNLYLGFITILGISTVLVSLLPVFQSPEFRVVRASLFFGMGFSGAAPILHKLIIFWDQPEALHTTGYEVLMGLLYGLGALVYATRIPERWMPGKFDIAGHSHQLFHVLVVAGAFTHYRAGLVYLKWRDIEGC
BrHHP4	Brara.A00124.1	MGGEAEIKQRLQPNAKGKRLWQKVKYQLVEYHALPAYLRDNEYILGHYRSEWPIKQILLSIFTIHNETLNVWTHLIGFFLFLALTIYTATKVPSVVDLHSLQHRLPDVLRKTDLHKLHSDLMSRLPSSPSKWHVMELLYNCLPERFSHGNNTDMCVLHSVKEDLANMIAPLIFRPITRWPFYAFLGGAMFCLLASSTCHLLSCHSERVSYIMLRLDYAGIAALISTSFYPPVYYSFMCDPFFCNLYLGFITILGISTVLVSLLPVFQSPEFRVVRASLFFGMGFSGAAPILHKLIIFWDQPEALHTTGYEVLMGLLYGLGALVYATRIPERWMPGKFDIAGHSHQLFHVLVVAGAFTHYRAGLVYLKWRDIEGC
BrHHP5	Brara.K01231.1	MGGEAEIKQQLQPKSLPAETIEKKQHNVKRRRLWQKVKYQLVEYHALPAYLRDNEYIIGHYRSEWPIKQILLSIFTIHNETLNVWTHLIGFFLFLSLTIYTATKVPSVVDLHSLQDRLPDILRKTDLHKLHSDLMSHLPSSPSKWHVMELLYNCLPERFSHGNYTDMCVLHSVKEDLANMIAPLIFRPITRWPFYAFLGGAMFCLLASSTCHLLSCHSERVSYIMLRLDYAGIAALIATSFYPPVYYSFMCDPFFCNLYLGFITTLGIATVLVSLIPVFQTPEFRVVRASLFFGMGFSGAAPILHKLIIFWDQPEALHTTCYEILMGLLYGLGALVYVTRIPERWMPGKFDIAGHSHQLFHVLVVAGAFTHYRAGLLYLKWRDIEGC
DcHHP5	DCAR_021464	MDNCEQEYRRKVGHRAESPKEKGKMLWKKVKYQLVEYHSLPAFLKDNEFILGHYRSEWPLKQIFFSVFSIHNETLNVWTHLIGFLLFLTLTIHTVMKIPYVVDLHKFENVREDLKTSLPLAHVLPSLSSWRSAKFLPNYIPEQFSQRNHSDVCALHSIKENVANTIAPVMVRPITRWPFFAFLGGAMFCLLASSMCHLLSCHSKRLSYIMLRLDYAGIATIISTSFYPPVYYSFMCNPFFCNLYLGFITLLGMGTIIGSLLPVFDRSEFRSIRASLFFAMGFSGVVPILHKLIMFWHQPEALHTTGYEVLMGSLYGLGALVYAMRVPERWIPGKVDIAGHSHQLFHILVLAGALTHYRAGLVYLKWRDLEGC
OsHHP4	LOC_Os03g13040.1	MAAAAGEEVEAARWAEAEDERKEGLRRRRRYGLVEYRALPGYMRDNEYILRHYRCEWPLPQVLLSAFSIHNETLNVWTHLIGFFIFLVLTIYTATQVPNVVDLQSLQHLPDVLRNADLHKIQTELVACLPSLPHLSDLQKLKDELKSSWNSIEVLPSLSRWHLLELLSSCLPHRFTHSNETSLSVLQSMKEDIANMIAPQLIRPIPRWPFYAFLGGAMFCLLASSTCHLLSCHSRRLAYIMLRLDYAGIAALIATSFYPPVYYSFMCYPFFCNLYLSCITILGVATIAFSLLPVFQNPEFRTIRACLFFGMGASGVIPVIHKLILFWHQPEALHTTAYEVLMGLFYGIGALVYATRVPERWMPGKFDIAGHSHQLFHVLVVAGAYTHYHSGLVYLKWRDVQGC
OsHHP5	LOC_Os12g32640.1	MEMMSLEEEETMASPTTSSCGTCKCGANDDKAKKMKTKTKKCELVGYEELPEWLKDNEFIHGYYRCEWPMKETILSIFSIHNETLNVWTHLIGFLLFLCLAIFTAMVIPSGDNLHSNSSRSRSNATAMDYYYIHGDLMVMSNMTRVLRHEALAAAACLLLHDPADLSQHEQISTSCPTNTSSCYTSSSSFSHLHNVRQHAIQDAGKVTAATAAAIAEPITRWPVFAYLGGAMACLLASTACHLLLCHSERANYVTLRLDYAGIAALIVASFLPIVHYSFLCDPWLRRAYTAAIACAGAATVTASLVPAFQSPRLRPLRAALFSGLAASGVVPVAHKMVLYGGTVREAATSARCEAAMGALYALGVAVYAARVPERWFPGRFDLVGHSHQLFHLLVVAGAYAHYLGALEYLKWRDAVKC
SlHHP4	Solyc02g092230.2.1	MGKGKEKLTGNGSREDNELLSPSKEGKGKRLWKKVKYQLVEYHSLPGYLKDNEYILGHYRAEWPLKQALLSIFTIHNETLNVWTHLIGFFLFLALTIYTATKVPKVVDLHSLQNLPDVLRKADLHKLQAELLTCLPSLPYMPDLHKLRDGLLRSPSNWHIIDLLHNCLPERFSHSNHTDVCVLRSVKEDVANILAPLLVRPITRWPFYAFLGGAMFCLLASSTCHLLSCHSERLSYIMLRLDYAGIAALISTSFYPPVYYSFMCYPFFCNLYLGFITLLGIGTILGSLLPVFQTPEYRVIRASLFFGMGLSGAVPILHKLVLFWHQPEALHTTGYELLMGIFYGIGALVYAMRVPERWMPGKFDIAGHSHQLFHVLVVAGAYTHYRAGLVYLRWRDLQGC
SlHHP5	Solyc03g043930.2.1	MSHQHDFIPVFPFLSPHFNSEILSGRIIFIQLSESPFFKSVFPFFQLMVQWKMGSDVEKLEEALNGSGSSETQPFRDSISKKEKQKRLWKKVNNQLVEYHSLPGYLKDNEFILGHYRCEWPVKQALLSVFTIHNETLNIWTHLIGFFLFLVLTIYTAKKVPDIVDLQTLQNLPEKLSKIDLHKLPADLLPCIPSLPNMPDLHKLRENLLQLLSNCLPDRFSHGNHTDVGVLHSVKDVANVIAPLMVKPITRWPFYAFLGGAMFCLLASSTCHLLCCHSERLSYVMLRFDYAGIAALISTSFYPPVYYSFMCYPFFLNLYLGFITVLGVGAMLASLLPVFQKPKFRSLRAGLFFGMGMSGVAPILHKLILYWNQPEALHTTGYEVLMGVLYGIGALIYALRVPERWMPGKFDIAGHSHNLFHVLVVAGAYTHYRAGLIYLQWRDQQGC
ZmHHP5	GRMZM2G380789_T01	MSSTVTLEKTTAIQSDGGRAGVAGSPKQAANRSPLLVAKKGAEGGAKEKARCCGRRCELVSYDKLPEFLKHNEFIVDHYRSEWPVKEALLSAFSIHNETINVWTHLIGFFVFLALTVCAATMVPTTEYESPHLALATSSSTGLTMTNITGNAMVLRSYSADDGAVMAMKALRNVSAAETAAAVLPAGAGRGRVARWPFYAYLCGAMFCLLMSSACHLLACHSEHASYVFLRLDYAGITGLIVTSFYPLVYYTFLCDPFYQALYLGFITVSGAAAVAVSLLPVFERPELRWARAGLFACMGMSGLVPIVHKMLVFGARPEALLTTGYEVAMGAFYLAGVVVYATRVPERWMPGRFDLAGHSHQLFHVLVIAGAYAHYLAGLVYLGWRDMEGC

**Table 3:** Sequences for virus-induced gene silencing test (VIGS).

Name of Corresponding Gene	Sequences (5′-3′)
*BcHHP5-Silencing*	ATGAAACCCAGCGTTGTTGATCTTCACTCGCTTCAGCACCG
	GTGCTGAAGCGAGTGAAGATCAACAACGCTGGGTTTCAT
*BcPDS-Silencing*	TTGAGGAACAACGAGATGCTGACATGGCCAGAGAAAATA
	ATTATTTTCTCTGGCCATGTCAGCATCTCGTTGTTCCTCAA

## Abbreviations

ABA	abscisic acid
ANOVA	analysis of variance
bp	base pair
CBF	c-repeat-binding factor
COR	cold-regulated
GFP	green fluorescent protein
HHP	Heptahelical protein
NJ	Neighbor-Joining
ORF	open reading frame
PDS	Phytoene desaturase
RT-qPCR	real-time quantitative polymerase chain reaction
SA	salicylic acid
TYMV-VIGS	turnip yellow mosaic virus-induced gene silencing
